# CO_2_ fixation in above-ground biomass of summer maize under different tillage and straw management treatments

**DOI:** 10.1038/s41598-017-17247-8

**Published:** 2017-12-04

**Authors:** Qianqian Feng, Jing Xu, Yayun Zhang, Xiaosha Li, Jiaojiao Xu, Huifang Han, Tangyuan Ning, Rattal Lal, Zengjia Li

**Affiliations:** 1 College of Agronomy, State Key Laboratory of Crop Biology, Shandong Key Laboratory of Crop Biology, Shandong Agricultural University, Tai’an, 271018 Shandong P.R. China; 2 Carbon Management and Sequestration Center, School of Environment and Natural Resources, The Ohio State University, Columbus, OH 43210 USA

## Abstract

This study was conducted to quantify the potential for CO_2_ fixation in the above-ground biomass of summer maize (*Zea mays* L.) under different tillage and residue retention treatments. The treatments were paired and included conventional tillage with straw removed (CT_0_), conventional tillage with straw retained (CT_S_), no-till with straw removed (NT_0_), no-till with straw retention (NT_S_), subsoiling with straw removed (SS_0_), and subsoiling with straw retained (SS_S_). The results indicated that NT_S_ and SS_S_ can enhance translocation of photosynthates to grains during the post-anthesis stage. SS_S_ showed the highest total production (average of 7.8 Mg ha^−1^), carbon absorption by crop (Cd) (average of 9.2 Mg C ha^−1^), and total C absorption (Ct) (average of 40.4 Mg C ha^−1^); and NT_S_ showed the highest contribution of post-anthesis dry matter translocation to grain yield (average of 74%). Higher CO_2_ emission intensity and CO_2_ fixation efficiency (CFE) were observed for straw retention treatments. In comparison with CT_S_, the mean CFE (%) over four years increased by 26.3, 19.0, 16.5, and 9.4 for NT_0_, SS_0_, NT_S_, and SS_S_, respectively. Thus, SS_S_ and NT_S_ systems offer the best options for removing CO_2_ from the atmosphere while enhancing crop productivity of summer maize in the North China Plain.

## Introduction

The rising atmospheric CO_2_ concentration is recognized as the primary cause of global climate change. In general, agricultural ecosystems have the potential to enhance carbon sequestration in the soil^1,2^. A significant part of soil C is present in cultivated soils that occupy about 35% of the global land surface, and agricultural management has the potential to be a powerful tool for climate change mitigation and to increase soil fertility through soil organic carbon (SOC) sequestration^3^. Thus, adopting agricultural best management practices (BMPs) can change SOC decomposition and CO_2_ emission^4,5^. Tillage is one of the most important agricultural management practices to impact crop production^6^ and alter SOC sequestration^2^ along with changing CO_2_ emission^7^. A primary management option to increase SOC storage therefore is to increase inputs of biomass-carbon (C) into the soil (i.e., retention of crop residues on the soil)^2^.

The global demand for food production is increasing because of an increase in world population. Therefore, a strong increase in crop yield is needed to feed the world^8^. Grain yield of maize (*Zea mays* L.) is affected by a complex interaction of CO_2_ absorption from the atmosphere through photosynthesis, use efficiency of radiation for the above-ground (shoot) biomass production, and the harvest index (Hi). Higher the CO_2_-use efficiency of a cropping system, greater is the C resource that can be captured into the above-ground biomass resulting in a higher grain yield. The C stored in the stem is the most important, especially at the post–anthesis stage because of its translocation from dry matter into the grain yield^9,10^. The natural capacity of crop growth being an important factor that affects CO_2_ fixation per plant, any increase in CO_2_ fixation by agricultural crops occurs through increase in dry weight of the biomass produced^11^. Therefore, a strategy of removing CO_2_ from the atmosphere is to grow plants thereby sequestering CO_2_ into biomass through photosynthesis, and converting it into SOC^12,13^.

Hence, traditional agricultural systems must adopt BMPs to enhance the potential of C sequestration in agricultural ecosystems. Most research has thus far been focused on SOC sequestration and change in SOC stocks. SOC sequestration in the crop production system provides a good indicator to evaluate C sequestration. While prior research has focused on enhancing C sequestration in soil, C stocks in the above-ground biomass of crops (e.g., maize, rice, wheat, and other grasses) is another pathway of C storage which has not been given the attention it deserves. Thus far, potential contribution of the above-ground biomass associated with agricultural practices has largely been overlooked^14,15^. Further, it is critical to find effective measures to increase C sequestration rate in the biomass of agro-ecosystems. While, a notable progress has been made in quantifying C stocks in forests^16,17^, knowledge of C stocks in agricultural ecosystems is scanty, and any empirical relationship between above-ground biomass allocation and C stocks is mostly unknown^18,19^.

Therefore, this research was designed on the hypothesis that the efficiency of CO_2_ fixation (CFE) in the above-ground biomass of summer maize can be manipulated through controlled tillage methods and residue retention. The overall aims of this study were to: (1) quantify changes in C stock in the above-ground dry matter as influenced by different soil tillage and straw management treatments, and (2) calculate CFE in the above-ground biomass as affected by tillage methods and straw retention. This study is designed to provide valuable information on choosing best conservation agricultural practices based on crop establishment and efficient C management systems, and for achieving sustainable and high productivity, C stocks, and improved soil health and quality.

## Methods

### Site description

The experiment was conducted on a brown loam soil, classified as an Udoll according to the U.S. soil taxonomy^20^, at the Agronomy Station of Shandong Agricultural University (36°10′19″N, 117°9′03″E) located in the North China Plain (NCP). The current experiment was conducted from 2011 to 2014 during the summer maize-growing seasons. During these years, the summer maize (Zhengdan 958) was planted at a rate of 66600 seeds ha^−1^ after the winter wheat (*Triticum aestivum* L) was harvested. The summer maize was planted at a spacing of 27 cm within and 60 cm between the rows. The characteristics of the surface soil (0–20 cm) were as follows: SOM content: 1.35%, total concentration of N: 1.3 g kg^−1^, total P: 13.6 g kg^−1^, rapidly available N: 91.6 mg kg^−1^, and rapidly available P: 15.1 mg kg^−1^. Summer maize was seeded on June 6, 8, 13, and 20, and was harvested on October 15, 18, 15, and 15 in 2011, 2012, 2013, and 2014, respectively. Base fertilizer applied at the seeding included N, P_2_O_5,_ and K_2_O at the rates of 120, 120, and 100 kg ha^−1^, respectively. In addition, 120 kg ha^−1^ of N was top-dressed during the jointing stage. The fertilizers were banded over the rows, and no irrigation was used during the summer maize-growing seasons.

### Experimental design

The experimental site was prepared using a randomized block design with three replications. Three tillage treatments were initiated in 2002 and included: no-till (NT), subsoiling (SS), and conventional tillage (CT). The designated tillage practices were performed each autumn after the harvest of maize. Each tillage system involved two straw management treatments. The straw retained (S) treatment involved mechanically chopping straw into 3–5 cm long pieces and mulched on the soil surface, shallowly incorporating it under NT_S_, incorporated into the soil by moldboard ploughing to a depth of 20 cm under CT_S_, and buried to a depth of 40 cm by subsoiling under SS_S_ treatment; The straw removed (0) treatment involved removing the above-ground part of plants is removed and leaving a stubble height of 5–10 cm. The NT_0_ plots did not undergo any disturbance, except for seeding drill, while CT_0_ plots underwent moldboard ploughing to a depth of 20 cm, and the SS_0_ plots underwent subsoiling to a depth of 40 cm. The plots were disked twice before planting. An NT planter was used to plant summer maize in all the treatments. The plot size for the main plots and subplots was 60 × 15 m and 15 × 15 m, respectively.

### Measurements

#### Yield and yield component

The grain yields of maize were determined in late September after maturity every year with samples from an area of 8 m^2^ in the central rows of each plot. Yield components were measured from each sample, including numbers of productive ear, grains per ear, and one thousand grain weight.

#### Dry matter accumulation and translocation

In the later growing stage of anthesis and maturity in 2013 and 2014, samples of 5 plants per plot were selected randomly and cut manually at the ground level to determine the above-ground dry matter accumulation every year. In the laboratory, samples were divided into vegetative organs (including leaves, stems, leaf sheaths, cobs, and bracts) and ears, and their weight noted after first exposing the samples to 105 °C for 0.5 h and thereafter to 85 °C until constant weight. Various parameters related to dry matter measurements were calculated according to Liu *et al*. (2016)^10^:

1 $${\rm{DMR}}={\rm{DM}}-{{\rm{DM}}}^{\ast }$$

where, DMR is the dry matter translocation (kg ha^−1^); DM is the dry matter of the vegetative organs at anthesis stage (kg ha^−1^); and DM* is the dry matter of the vegetative organs (excluding grains) at maturity stage (kg ha^−1^). The respiration and root dry matter translocation were not taken into account in this equation.

2 $${\rm{DMRE}}=({\rm{DMR}}/{\rm{DM}})\times 100$$

where, DMRE is the efficiency of dry matter translocation (%).

3 $${\rm{CDMRG}}=({\rm{DMR}}/{\rm{GY}})\times 100$$

where, CDMRG is the contribution of post-anthesis dry matter translocation to grain yield (%); GY is the grain yield (kg ha^−1^).

#### Plant carbon translocation

The C absorption rate at specific growing stages of the summer maize was calculated according to Li *et al*. (2000)^21^:

4 $${{\rm{C}}}_{{\rm{d}}}={{\rm{C}}}_{{\rm{f}}}\times {{\rm{D}}}_{{\rm{w}}}={{\rm{C}}}_{{\rm{f}}}\times {{\rm{Y}}}_{{\rm{w}}}/{{\rm{H}}}_{{\rm{i}}}$$

where, C_d_ is the C absorption by summer maize at the specific growing stage; C_f_ is the C source efficiency for synthesizing dry matter (0.4709); Y_w_ is the economical yield; D_w_ is the organic yield; H_i_ is the index (0.40).

Following the summer maize assimilation of CO_2_ and release of O_2_ into the air through photosynthesis, carbohydrates were synthesized during the growth. Therefore, the amount of the absorbed C can be calculated with the increase in the weight of dry matter. The total C stock for the summer maize growth (Ct) was calculated by using Eq. 5:

5 $${\rm{Ct}}=\sum {\rm{Cd}}$$

where, Ct is the total C stock during the entire summer maize growth season; and Cd is the C absorption of the summer maize at specific growing stage.

#### CO_2_ emission

The rate of soil respiration was measured using an LI-8100 soil CO_2_ flux system with static chambers made of polyvinyl pipe for obtaining samples of soil air. The gas chamber was 15.7 cm in height and 25.0 cm in diameter, and was inserted tightly into the ground between the rows without removing any of the surface soil. A sample of the CO_2_ evolved was obtained and analyzed five times during each growing period. Each sampling lasted for less than 2 min on a sunny day between 09:00 and 10:00 am.

The intensity of CO_2_ emission per grain yield (Mg CE Mg^−1^) was calculated based on the relationship of grain yield per cumulative CO_2_ emission at maturity. The cumulative CO_2_ emission was calculated by using the method of Liu^15^ as shown in Eq. 6:

6 $${\rm{M}}=({{\rm{F}}}_{{\rm{i}}+1}+{{\rm{F}}}_{{\rm{i}}})/2\times ({{\rm{t}}}_{{\rm{i}}+1}-{{\rm{t}}}_{{\rm{i}}})\times 24$$

where, M is the cumulative emission of CO_2_ (mg cm^−2^); F is the soil surface CO_2_ flux (kg CO_2_ ha^−1^ h^−1^); i is the sample number; and t is the number of days after sowing.

#### CO_2_ fixation efficiency

CO_2_ fixation efficiency (CFE; Mg yield CE Mg^−1^) is evaluated by CO_2_ use efficiency per grain yield that is equivalent to the emitted CO_2_ that was fixed by grain yield and is calculated as:

7 $${\rm{CFE}}={\rm{Y}}/{\rm{CF}}$$

where, Y is the grain yield of the maize (Mg), which was measured at maturity on an area of 8 m^2^ corresponding to the central rows of each plot; and CF is the CO_2_ flux per unit (Mg CE ha^−1^).

### Data analysis

The experimental results are presented as means and standard deviations. Multiple comparisons were made using the least significant difference (LSD) test at *p* = 0.05 level. The data were statistically analyzed using analysis of variance (ANOVA) through the SPSS statistical analysis package (Version 13.0) at *p* = 0.05 level of significance.

## Results

### Yield and yield compositions

Tillage methods and straw retention significantly influenced the summer maize ear grain number (*p* < 0.05), which under NT_S_ treatment was less than that under CT_S_ and SS_S_ treatments (Table 1). Straw retention increased summer maize 1000-grain weight and the ear grain number. However, no significant difference in the number of productive ears was observed among different treatments. During the four years, SS_S_ had the highest production (average: 7.8 Mg ha^−1^). The grain yield under different treatments in 2011 followed the order of SS_S_ > CT_S_ > NT_S_, wherein the grain yield under the SS_S_ treatment was 5.1% more, and that under NT_S_ was significantly decreased by 2.1% lower (*p* < 0.01) than that under CT_S_ treatment. Analyses of the data for 2011 and 2012 for tillage methods showed that the production for CT_S_, NT_S_, and SS_S_ treatments were 6.0, 6.0, and 6.2 Mg ha^−1^, respectively. The SS_S_ treatment significantly increased the yield of summer maize (*p* < 0.05), and the ratio of yield increase was 2.2%. There was no significant difference in maize yield between NT_S_ and CT_S_ (*p* > 0.05). In addition, the crop production in treatments without and with residue retention was on average 4.2 and 4.7 Mg ha^−1^, respectively, indicating average yield increase of 11% with residue retention. The production under NT_0_ treatment was the lowest in all the four years of the study. It was 7.0 and 6.9 Mg ha^−1^ in 2011 and 2012, respectively, and declined further in 2013 and 2014.

**Table 1:** Effects of tillage methods and straw retention on grain yield and yield components for summer maize (2011–2014).

Year	Treatment	1000-grain weight (g)	Grain number per ear (grain ear^−1^)	Number of productive ear (10^4^ ear ha^−1^)	Yield (Mg ha^−1^)	Relative yield (%)
2011	CT_0_	312.6d	521.8b	6.7a	7.0d	1.0
	CT_S_	321.0a	525.5a	6.7a	7.8b	1.1
	NT_0_	311.3d	513.3d	6.7a	7.0d	1.0
	NT_S_	319.9ab	518.0c	6.7a	7.7b	1.1
	SS_0_	317.3c	519.9c	6.7a	7.2c	1.0
	SS_S_	320.5a	525.5a	6.7a	7.9a	1.1
2012	CT_0_	313.7d	529.0b	6.7a	7.1c	1.0
	CT_S_	319.0ab	533.6a	6.7a	7.8ab	1.1
	NT_0_	309.5e	514.3d	6.7a	6.9d	1.0
	NT_S_	317.4b	529.9b	6.7a	7.9a	1.1
	SS_0_	316.0c	529.0b	6.7a	7.1c	1.0
	SS_S_	319.8a	528.1b	6.7a	7.9a	1.1
2013	CT_0_	298.8g	549.0a	6.7a	6.4e	0.9
	CT_S_	327.4b	514.1b	6.7a	7.4c	1.1
	NT_0_	326.8b	468.2d	6.7a	7.1d	1.0
	NT_S_	327.7b	482.2d	6.7a	7.4c	1.1
	SS_0_	311.0f	513.1b	6.7a	7.7b	1.1
	SS_S_	330.0a	499.3c	6.7a	8.0a	1.1
2014	CT_0_	312.7d	387.2f	6.7a	5.5e	0.8
	CT_S_	359.7b	481.1b	6.7a	7.0c	1.0
	NT_0_	329.9c	487.3a	6.7a	6.4d	0.9
	NT_S_	334.4c	446.2c	6.7a	7.0c	1.0
	SS_0_	310.8d	442.9c	6.7a	7.3b	1.1
	SS_S_	349.1a	384.4f	6.7a	7.4a	1.1

CT_0_ represent conventional tillage with straw removed, CT_S_ represent conventional tillage with straw retained, NT_0_ represent no-till with straw removed, NT_S_ represent no-till with straw retained, SS_0_ represent subsoiling with straw removed, and SS_S_ represent subsoiling with straw retained. a, b, and c are ± standard errors of means (n = 3).

Based on the production under CT_0_ in 2011, the relative production was calculated for the other years. Results showed that most treatments with straw retention had a ratio of >1. In comparison with the yield under CT_0_ treatment in 2011, relative production under SS_S_ was increased by 13.0, 14.0, 14.0, and 6.0% in 2011, 2012, 2013, and 2014, respectively.

### Dry matter translocated from vegetative organs to grain after anthesis

After the anthesis of summer maize, translocation efficiency of dry matter from the vegetative organs ranged from 8.0–32.9% in 2013, and 8.0–37.8% in 2014 (Table 2). The contribution of dry matter translocation to grain yield ranged from 15.3–58.9% in 2013 to 14.0–89.4% in 2014.

**Table 2:** The amount of dry matter translocation from vegetative organs to grain and the accumulation amount after anthesis.

Year	Treatment	DM (g)	DM*(g)	DMR (g)	DMRE (%)	CDMRG (%)
2013	CT_0_	119.7bc	89.3c	30.4b	25.0b	47.7b
	CT_S_	114.4c	99.3b	15.1d	13.1d	20.4d
	NT_0_	126.1b	84.6c	14.0d	12.4d	19.0e
	NT_S_	115.0c	101.0b	41.5a	32.9a	58.9a
	SS_0_	122.8b	100.1b	22.8c	18.7c	29.5c
	SS_S_	137.3a	125.1a	12.2d	8.0e	15.3e
2014	CT_0_	125.4c	94.6c	30.8c	24.6c	56.3c
	CT_S_	141.5b	111.2a	30.3c	21.4c	43.6d
	NT_0_	137.3b	92.9c	44.5b	32.4b	69.7b
	NT_S_	164.6a	102.3b	62.2a	37.8a	89.4a
	SS_0_	124.7c	100.1b	24.7d	19.8d	33.7e
	SS_S_	128.8c	118.5a	10.4e	8.0e	14.0f

CT_0_ represent conventional tillage with straw removed, CT_S_ represent conventional tillage with straw retained, NT_0_ represent no-till with straw removed, NT_S_ represent no-till with straw retained, SS_0_ represent subsoiling with straw removed, and SS_S_ represent subsoiling with straw retained. DM represent dry matter at post-anthesis period, DM* represent dry matter at maturity, DMR represent dry matter translocation, DMRE represent dry matter translocation efficiency, CDMRG represent contribution of dry matter translocation to grain yield. (1) Means of 5 stem per pot; (2) Different letters of a-e mean significant differences at 5% level.

The amount (DMR), efficiency (DMRE), and contribution to grain yield (CDMRG) of dry matter translocation from the vegetative organs to grains was the highest under NT_S_ and the lowest under SS_S_. However, dry matter after the anthesis and maturity stage of aerial parts per unit area in two years was the highest under SS_S_ treatment, indicating the high potential to improve the transport efficiency under SS_S_.

### Carbon absorption

The data in Table 3 show the significant effects of tillage methods and straw retention on C absorption by maize and total carbon absorption during the summer maize growing season.

**Table 3:** Different treatments on total carbon conversion (Mg C ha^−1^).

Treatment Year	CT_0_	CT_S_	NT_0_	NT_S_	SS_0_	SS_S_
**Cd**_**m**_ **(2011–2014) Carbon absorption of summer maize at mature stage**						
2011	8.2e	9.1b	8.2e	9.0c	8.4d	9.3a
2012	8.4c	9.2b	8.1d	9.3a	8.4c	9.3a
2013	7.5e	8.7c	8.3d	8.7c	9.1b	9.4a
2014	6.4e	8.2c	7.5d	8.2c	8.6b	8.7a
**Ct (2013–2014) Total Carbon absorption**						
2013	31.0d	36.6c	39.9b	36.3c	36.8c	41.5a
2014	32.9e	39.1a	37.4b	36.0cd	36.7c	39.3a

CT_0_ represent conventional tillage with straw removed, CT_S_ represent conventional tillage with straw retained, NT_0_ represent no-till with straw removed, NT_S_ represent no-till with straw retained, SS_0_ represent subsoiling with straw removed, and SS_S_ represent subsoiling with straw retained. a, b, and c are ± standard errors of means (n = 3).

The C absorption by summer maize at maturity stage (Cd_m_) in four years from 2011 to 2014 and total C absorption (Ct) in two years were the highest under SS_0_ and SS_S_ treatments, and the average absorption increased by 20.3% compared to the lowest under CT_0_ treatment. The total C absorption was increased by 33.8% and 19.5% under SS_S_ compared to CT_0_ in 2013 and 2014, respectively. Our results indicate that under the SS_S_ treatment, not only was the absorption of C high, but translocation of C from stem to grain was more, implying that this treatment was efficient for carbon transfer contributing to efficient C management.

### Carbon dioxide (CO_2_) emission intensity

**Figure 1:**
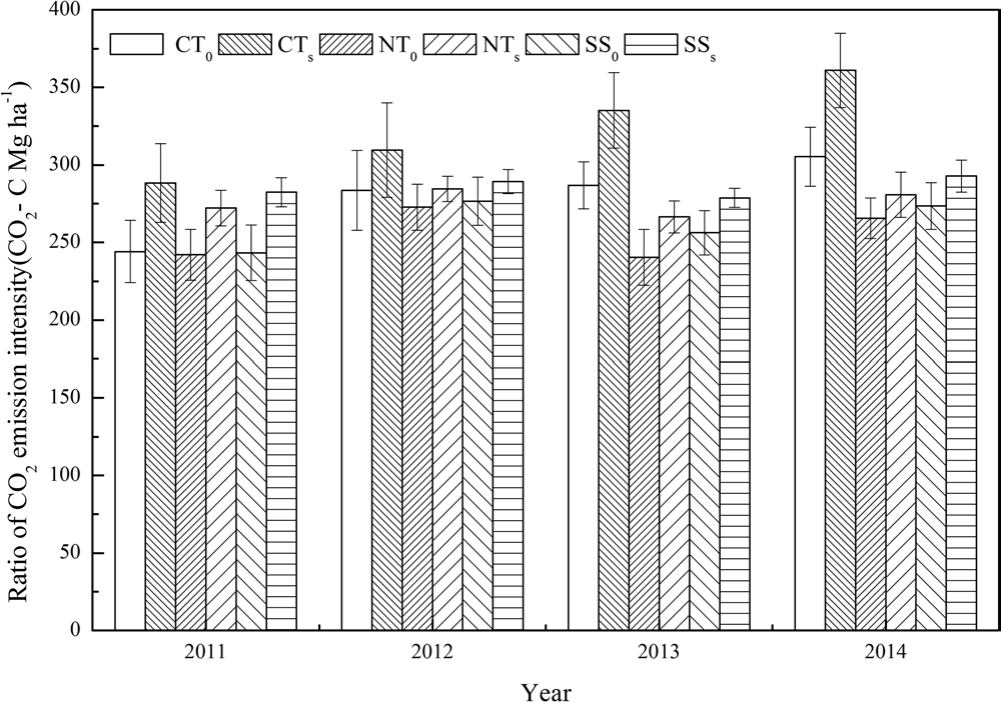
Effects of tillage methods and straw retention on the ratio of CO_2_ emission intensity per grain yield in summer maize growing seasons 2011–2014 (CO_2_-C Mg ha^−1^). CT_0_ represent conventional tillage with straw removed, CT_S_ represent conventional tillage with straw retained, NT_0_ represent no-till with straw removed, NT_S_ represent no-till with straw retained, SS_0_ represent subsoiling with straw removed, and SS_S_ represent subsoiling with straw retained. Bars are ± standard errors of means (n = 3).

CO_2_ emissions intensity can be estimated with the CO_2_ emission per unit of maize grain yield, a high value of which depends almost exclusively on soil management (Fig. 1). The CO_2_ emission intensity under CT_S_ treatment was the highest, and was significantly higher under straw retention treatments than under straw removed treatments. These results indicate that straw retention treatments significantly increased C emission, which significantly enhanced soil respiration intensity. Among the three methods of tillage, CO_2_ emission intensity was the lowest under NT_S_, medium under SS_S_, and the highest under CT_S_.

### CO_2_ fixation efficiency per grain yield (CFE)

**Figure 2:**
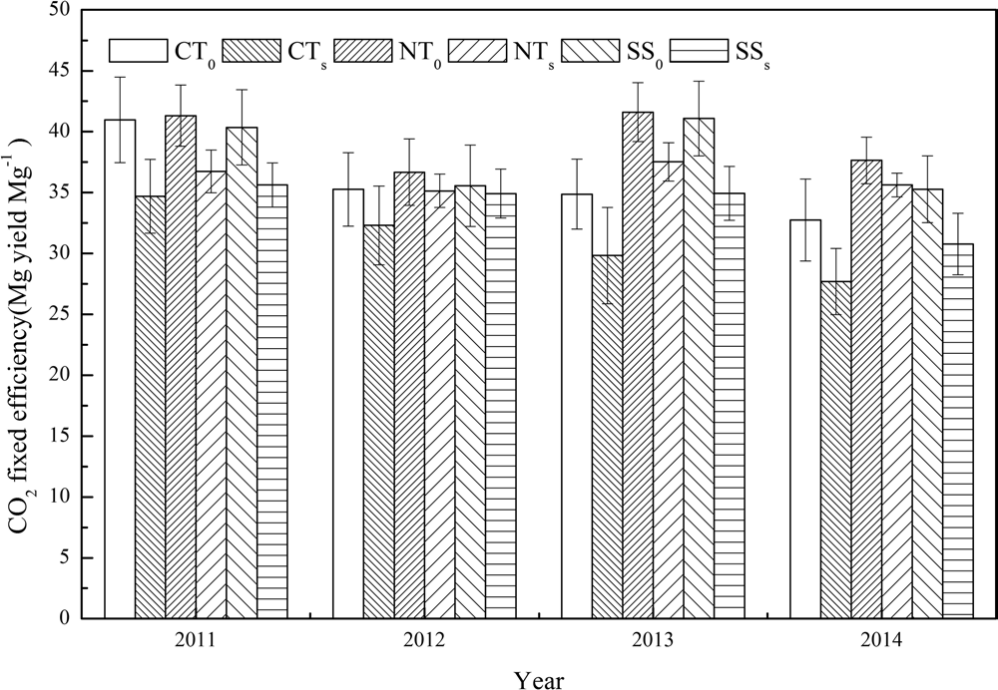
Effects of tillage methods and straw retention on CO_2_ fixation efficiency (CFE) per grain yield during the summer maize growing seasons 2011–2014 (Mg yield Mg^−1^ CE). CT_0_ represent conventional tillage with straw removed, CT_S_ represent conventional tillage with straw retained, NT_0_ represent no-till with straw removed, NT_S_ represent no-till with straw retained, SS_0_ represent subsoiling with straw removed, and SS_S_ represent subsoiling with straw retained. Bars are ± standard errors of means (n = 3).

CFE was significantly higher under straw removed than under the straw retained treatments, and was significantly higher under NT_S_ and SS_S_ than under CT_S_ treatment (Fig. 2). Thus, the CFE under NT_0_ (average of 39.3 Mg yield Mg^−1^ CE) was the highest among all treatments. The average CFE was 34.1 Mg yield Mg^−1^ CE under SS_S_, and 36.3 Mg yield Mg^−1^ CE under NT_S_. While the straw retention significantly increased soil C emission and the CO_2_ fixation efficiency, the use of NT and SS with straw retention is a priority for in-depth research.

## Discussion

In cereals and particularly in maize production, post-anthesis assimilation can enhance and stabilize crop yield. C assimilates available for grain production is determined by the C assimilated during the grain-filling period plus the assimilated reserves stored in the stem and leaves. The effects of conservation tillage and straw retention on dry matter accumulation have been widely researched. For example, Huang *et al*. (2009)^22^ showed that SS and straw mulching increased the total dry matter accumulation after anthesis, and enhanced the translocation of dry matter into the grains. However, it is well established that tillage methods can modify the soil environment, improve porosity, exacerbate disintegration of aggregates, mix plant materials deeper into the soil, and thereby increase crop biomass and soil contact. The data presented herein shows that the rate of contribution of vegetative organs to grain yield after the anthesis increased by 22.1% under NT with residue retention when compared with that under CT_0_. The DMR, DMRE, and CDMRG were highest under NT_S_ and lowest under SS_S_ in 2013 and 2014. However, the DM and DM* were highest under SS_S_, indicating that there exists a large potential for dry matter transport efficiency under SS_S_ treatment.

Lal land Kimble^23^ argued that conservation tillage is a recommended measure to improve SOC in agricultural ecosystems. In the surface soil layer, SOC content under NT is higher than that under CT and reduced tillage^24^. Straw retention can also increase the rate of soil CO_2_ emission, which also increases the proportion of CO_2_ emission per grain yield. The data presented herein show that NT could significantly reduce the CO_2_ emission per grain yield^7,14^. Further, the analysis of Cd and Ct showed that SS_S_ treatment not only absorbed a large amount of C, but also transported more C into the grain yield. Several studies have suggested that, Maize [*Zea mays* L.], a C_4_ plant, is less sensitive to elevated CO_2_ than C_3_ plants^25–27^; however, there are also some research evidenced that elevated CO_2_ increases both above- and below ground biomass in maize, showing increases in yield^19,28^, one possible result of summer maize grain yield stimulation by elevated CO_2_ is maybe “air-fertilizer” effect of an enhancement of plant height, leaf area and above ground biomass^29,30^. Under water stress, crop responses to CO_2_ were not sensitive probably because high CO_2_ reduced stomata conductance^25^, which resulted in low photosynthetic rate. However, in North China, summer maize growing season is right the rainy season, water is not a limited factor for the crops growth, this maybe one reason why the absolute increase in summer maize grain yield in response to elevated CO_2_. However, further studies are still needed. In our study, with different tillage and straw treatments, the CO_2_ concentrations were different, and hence, CO_2_ fixation in the summer maize above-ground biomass was different. As a result, grain yield of summer maize was different, and among them SS_S_ had the highest total production during the four years.

While comparing CFE values across the amount of CO_2_ fixed, differences between treatments were small; the largest difference in CFE values was observed for the NT_0_ which was significantly higher and the CT_S_ which was significantly lower than that for other treatments. Thus, CFE is affected by various biotic and abiotic factors^31,32^. Future studies should be conducted to simultaneously measure the effects on both C stock and the turnover rate of microbial biomass by using ^13^C labeling methods, to better understand and predict the effects of tillage methods and straw retention on CFE under a long-term soil C sequestration. Such studies will contribute to meeting the challenges of global climate change.

The net anthropogenic CO_2_ emissions must be reduced to mitigate climate change. One of the solutions to mitigate climate change depends on conserving as much crop biomass as possible^11^. The large production of agricultural above-ground biomass not only offers an opportunity to mitigate anthropogenic climate change but also improves food security while improving the environment.

Photosynthesizing atmospheric CO_2_ into maize dry matter comprising of the above-ground biomass and grain yield is a viable option to address the global priorities of ensuring food security and counteracting CO_2_ emission into the atmosphere.

The data presented herein indicate that SS and NT along with retaining crop residues can optimize the CO_2_ fixation capacity of above-ground biomass and could be the preferred BMPs for mitigating anthropogenic climate change. Thus, with reference to food demand and climatic scenarios^8^, adopting such BMPs in agro-ecosystems is a triple-win option. And experiments conducted in various kinds of soils and different regions are needed to confirm our findings at a larger spatial scale.

## Conclusion

There is no simple solution to improve atmospheric chemistry and increase crop productivity while enhancing resource use efficiency and protecting environmental quality. The present study shows that SS_S_ and NT_S_ treatments not only absorb a large amount of C, but also transport more C into summer maize grains. The mean summer maize grain yield for SS_S_ and NT_S_ were the highest over four years. Overall, our results indicate that SS_S_ and NT_S_ optimized summer maize grain yield and Cd in the brown soil in the North China Plain.
